# Silica Nanoparticles Provoke Cell Death Independent of p53 and BAX in Human Colon Cancer Cells

**DOI:** 10.3390/nano9081172

**Published:** 2019-08-16

**Authors:** Susanne Fritsch-Decker, Zhen An, Jin Yan, Iris Hansjosten, Marco Al-Rawi, Ravindra Peravali, Silvia Diabaté, Carsten Weiss

**Affiliations:** Institute of Toxicology and Genetics, Karlsruhe Institute of Technology, Hermann-von-Helmholtz-Platz 1, 76344 Eggenstein-Leopoldshafen, Germany

**Keywords:** synthetic amorphous silica, nanoparticles, colon cells, in vitro toxicity, cell death

## Abstract

Several in vitro studies have suggested that silica nanoparticles (NPs) might induce adverse effects in gut cells. Here, we used the human colon cancer epithelial cell line HCT116 to study the potential cytotoxic effects of ingested silica NPs in the presence or absence of serum. Furthermore, we evaluated different physico-chemical parameters important for the assessment of nanoparticle safety, including primary particle size (12, 70, 200, and 500 nm) and surface modification (–NH_2_ and –COOH). Silica NPs triggered cytotoxicity, as evidenced by reduced metabolism and enhanced membrane leakage. Automated microscopy revealed that the silica NPs promoted apoptosis and necrosis proportional to the administered specific surface area dose. Cytotoxicity of silica NPs was suppressed by increasing amount of serum and surface modification. Furthermore, inhibition of caspases partially prevented silica NP-induced cytotoxicity. In order to investigate the role of specific cell death pathways in more detail, we used isogenic derivatives of HCT116 cells which lack the pro-apoptotic proteins p53 or BAX. In contrast to the anticancer drug cisplatin, silica NPs induced cell death independent of the p53–BAX axis. In conclusion, silica NPs initiated cell death in colon cancer cells dependent on the specific surface area and presence of serum. Further studies in vivo are warranted to address potential cytotoxic actions in the gut epithelium. The unintended toxicity of silica NPs as observed here could also be beneficial. As loss of p53 in colon cancer cells contributes to resistance against anticancer drugs, and thus to reoccurrence of colon cancer, targeted delivery of silica NPs could be envisioned to also deplete p53 deficient tumor cells.

## 1. Introduction

Synthetic amorphous silica (SAS) nanoparticles (NPs) are produced in large amounts for applications in industry and medicine. Many consumer products including food contain SAS, which occurs in different forms depending on the process of its manufacture. Food-grade SAS (E551) includes fumed (pyrogenic) silica and hydrated silica (precipitated silica, silica gel, and hydrous silica). Colloidal silica (silica sol) is not authorized as a food additive [1], but is an emerging material for various biomedical applications such as drug delivery [2]. Addition of SAS to food products has, for example, stabilizing, anti-caking, anti-settling, and emulsifying effects [3].

SAS food additive has been used for decades and is considered to be safe for consumers. Yang et al. [4] reported the occurrence of SAS at 1.3–16.3 mg Si/g dry food product, mainly in processed food such as coffee creamer, pudding powder, cake mix, or in probiotic tablets. In all exposure scenarios studied by EFSA (European Food Safety Authority), the lowest exposure was reported in the elderly, while the highest was in infants and in children, ranging from 3.9 to 74.2 mg/kg body weight per day and from 8.4 to 162.7 mg/kg body weight per day, respectively [1].

The effects of oral uptake of food-grade SAS nanoparticles have been reviewed recently [1,3,5,6,7,8]. In vivo studies with rats found no acute toxicity due to SAS ingestion, but fibrosis in the liver at high dosages [9]. In a study with mice, upon oral exposure to SAS NPs, increased pro-inflammatory cytokine levels (IL-1β, IL-6, and TNF-α) were detected in the colons. Additionally, ingested SAS NPs increased the richness and diversity of microbial species within the intestinal tract [10]. Recently, Siemer et al. found that nanomaterials including SAS impact the (patho)biology of bacteria occurring in the gut [11].

In addition, in vitro studies suggest that food-grade SAS is potentially hazardous. In particular, fumed SAS is more toxic than previously assumed. The study of Zhang et al. [12] showed that fumed silica was more active in producing ROS (Reactive Oxygen Species) and causing red blood cell hemolysis. In Caco-2 cells, fumed SiO_2_ NPs induced cytotoxicity, DNA damage, and glutathione (GSH) depletion in serum-free medium [13,14]. Using human HT29 colon cells, it has been demonstrated that silica NPs are taken up into cells and stimulate cell proliferation under high-serum conditions. Under low-serum-conditions, cytotoxic effects were observed [15]. The study also found that SiO_2_-NPs enhanced the biosynthesis of GSH via the mitogen activated protein kinase (MAPK) pathway and caused oxidative DNA damage. Activation of the MAPK pathway and cytotoxicity in response to silica NPs were also observed in the human stomach cell line GXF252L [16].

Winter et al. found that SiO_2_-NPs affected the viability of murine dendritic cells and activated the inflammasome, suggesting that oral administration of these NPs could promote intestinal inflammatory responses [17]. More recently, the Toll-like receptor signaling pathway was demonstrated to be upstream of the inflammasome, and is essential in murine dendritic cells for the induction and release of IL-1β in response to food-grade nanosilica [18].

Before the particles reach the cells of the intestinal mucosa, they must pass the gastrointestinal tract, encompassing the oral cavity, esophagus, stomach, and intestine, and are exposed to different chemical conditions. The fluids in the gastrointestinal lumen contain complex mixtures of biomolecules such as digestive enzymes and food in different stages of digestion. Furthermore, osmotic concentration, pH, and the gut microbiome change during the passage through the gastrointestinal tract. All these parameters affect the ingested NPs and can alter the physico-chemical properties of the particles, which may influence the toxicological outcome [19]. Therefore, it is very difficult or nearly impossible to mimic all the parameters and physiological conditions encountered in vivo in an in vitro experiment. Furthermore, it has been demonstrated that biomolecules, in particular, proteins, bind to the particle surface and form a so-called biomolecular corona [20,21], which has consequences for the biological activity of nanomaterials [22,23]

The epithelial barrier of the gut represents a target for potentially cytotoxic effects of ingested silica NPs. As in the absence of a protective protein corona silica NPs induce cell death, for example, in lung epithelial and phagocytic cells, reminiscent of apoptosis [24,25], we wanted to investigate the role of specific cell death pathways in intestinal epithelial cells in more detail. Therefore, we used the human colon epithelial cancer cell line HCT116 as a model to evaluate the cytotoxic effects of SiO_2_-NPs as a function of different physico-chemical parameters important for the assessment of nanoparticle safety [26,27], i.e., concentration (10, 25, 50, and 100 μg/mL), duration of exposure (5, 24, and 48 h), particle size (12, 70, 200, and 500 nm), surface modification (–NH_2_ and –COOH), and protein coating with fetal bovine serum (FBS). Besides the wild-type cells, we used their isogenic derivatives, which lack the p53 or bax gene [28], to clarify the role of these pro-apoptotic factors for SiO_2_-NP-induced cell death.

## 2. Materials and Methods

### 2.1. Materials

Materials and reagents were obtained from the following suppliers: Dulbecco’s modified Eagle’s medium (DMEM) (cat no 41966), cell culture medium supplements, Hank’s balanced salt solution (HBSS, cat no 14025), Dulbecco’s phosphate-buffered saline (DPBS, cat no 14190094), BCA (bicinchoninic acid) protein quantitative assay kit (cat no 23225): ThermoFisher Scientific (Dreieich, Germany); fetal bovine serum (FBS),Lactate dehydrogenase (LDH)cytotoxicity detection kit (cat no 11644793001), Hoechst 33258 (Hoechst, cat no B2261), propidium iodide (PI, cat no P4170), CDDP [cisplatin, *cis*-diamineplatinum (II) dichloride, cat no P4394], chemicals for sodium dodecylsulfate polyacrylamide gel electrophoresis (SDS-PAGE), and standard laboratory chemicals: Sigma-Aldrich (Taufkirchen, Germany); AlamarBlue reagent: AbD Serotec (Puchheim, Germany, cat no BUF012B); Aerosil^®^ 200 (SiO_2_—12 nm): Evonik Industries (Frankfurt am Main, Germany); Stöber-synthesized silica SiO_2_—70 nm cat no 43-00-701, SiO_2_-NH_2_—70 nm cat no 43-01-701, SiO_2_-COOH—70 nm cat no 43-02-701, SiO_2_—200 nm cat no 43-00-202, SiO_2_—500 nm cat no 43-00-502: Micromod Partikeltechnologie (Rostock, Germany). All particles are listed in Table 1.

Enhanced chemiluminescence (ECL) reagents were obtained from GE Healthcare Amersham (cat no 2232). Antibodies for detection of cleaved caspase 3 (Asp 175, cat no 9661), caspase 8 (1C12, cat no 9746), and cleaved caspase 9 (Asp 315, cat no 9505) were purchased from Cell Signaling Technology (Frankfurt am Main, Germany). Antibodies against p53 (DO-1, cat no sc-126) and PCNA (PC-10, cat no sc-56) were from Santa Cruz (Heidelberg, Germany). Secondary horseradish-peroxidase (HRP)-conjugated antibodies: DAKO (Hamburg, Germany), pan-caspase inhibitor Q-VD-OPh: 5-(2,6-Difluorophenoxy)-3-[[3-methyl-1-oxo-2-[(2-quinolinylcarbonyl)amino]butyl]amino]-4-oxo-pentanoic acid hydrate: MP Biomedicals, Heidelberg, Germany, cat no 03OPH10901.

### 2.2. Particle Suspensions and Characterization

For treatment of cells, the particle stock suspensions were generated just before preparing the diluted particle suspensions in cell culture medium with or without serum, which were added to the cells. Aerosil^®^ 200 NPs delivered as powder were suspended in sterile deionized water at 1 mg/mL, shortly vortexed and probe sonified with 15 strokes, 15% cycle duty, output control 5 (Branson Sonifier 250, Schwäbisch Gmünd, Germany). The other colloidal silica particles, which were delivered as suspension, were diluted to 1 mg/mL in deionized water and vortexed. These stock solutions were further diluted in medium to the desired concentrations.

For analysis by dynamic light scattering (DLS), NPs were further diluted to 50 µg/mL in deionized water or DMEM with or without serum. The samples were then analyzed directly after vortexing using the Zetasizer Nano ZS (Malvern Instruments Ldt., Herrenberg, Germany) at 25 °C.

For analysis by transmission electron microscopy (TEM), the particle suspensions in water were transferred onto TEM grids (Plano, SF162-6), dried, and analyzed by a Zeiss electron microscope (Zeiss 109T, Oberkochen, Germany) [29].

### 2.3. Calculation of the Effective Density and the Deposited Dose

To compare the effects of differently sized SiO_2_ particles, it was necessary to calculate the real dose deposited onto the cells over 24 h, as already described previously [30]. Briefly, the effective density of the particles in the respective medium was determined by the volumetric centrifugation method (VCM) according to Deloid et al. [31] at the highest applied concentration of 100 µg/mL. 1 mL of the particle suspension was filled into a packed cell volume (PCV) tube (TPP Techno Plastic Products, Trasadingen, Switzerland) and centrifuged in a swinging bucket rotor at 3.000 *g* for 1 h to collect the agglomerates in the capillary section of the tube. The volume of the pellet was determined using a measuring device from TPP Techno Plastic Products (Trasadingen, Swizerland). The effective density was then calculated according to the formula given in Deloid et al. [32]. The relative in vitro dose (RID) was determined by calculating the particle mass deposited onto the cell surface after 24 h using the distorted grid (DG) nanotransport simulator [33], based on hydrodynamic size (Table S1), effective density (Table S2), and other parameters in the respective media. 

### 2.4. Cells

HCT116 wt, p53^−/−^, and BAX^−/−^ cells (kindly provided by B. Vogelstein, John Hopkins University, Baltimore, MD, USA) were cultured as described before [34]. Briefly, the cells were maintained in Dulbecco’s modified Eagle’s medium (DMEM) supplemented with 10% fetal bovine serum (FBS), 100 U/mL penicillin and 100 mg/mL streptomycin at 37 °C in a humidified atmosphere containing 5% CO_2_. Medium was changed every 2 days. Control cells were treated with medium alone or with 0.1% DMSO, the solvent used for the caspase inhibitor.

### 2.5. Cell Death Analysis by Fluorescence Microscopy 

For detection of cell number and stages of cell death by automated microscopy, 8000 cells were seeded per well of a 96 well plate. On the next day, the cell culture medium was discarded and the cells were treated according to the experimental design. After the incubation period, analysis was performed as previously described [30]. Briefly, Hoechst 33342 and propidium iodide (PI) were added to a final concentration of 0.3 µg/mL and 0.5 µg/mL, respectively. After 30 min incubation in the dark, bright field (BF) and fluorescence images were acquired from four positions in the well using an automated Olympus IX81 fluorescence microscope and a 10× objective (Olympus, Hamburg, Germany). The Hoechst dye was detected at excitation and emission wavelengths of 350 and 450 nm, respectively. PI dye was detected at 488 nm and 590 nm, respectively. The images were analyzed by the scan^R analysis software (version 2.7.3, Olympus, Hamburg, Germany) to obtain the total number of cells (Hoechst channel) and the number of early apoptotic, late apoptotic, and necrotic cells (combination of Hoechst and PI channel), as described previously [30].

### 2.6. Real-Time Imaging at the Single Cell Level

For real-time imaging at single cell level, HCT116 cells were first seeded in 96 well plates, as described above, and incubated overnight. Real-time imaging was performed as published previously [25]. Briefly, before treatment with particles, cells were stained with 0.1 μg/mL Hoechst and 0.083 μg/mL PI for 1 h at 37 °C and 5% CO_2_, followed by an incubation with SiO_2_ NPs over 24 h in a microscope incubator box (EMBLEM, Heidelberg, Germany) under control of CO_2_, humidity, and temperature (37 °C, 5% CO_2_). Two images per well and channel (bright-field, Hoechst and PI) were acquired using the automated fluorescence microscope IX81 (Olympus, Hamburg, Germany) with a 20-fold objective. The NIH ImageJ Software (version 1.50b, Bethesda, MD, USA) was used to convert images to videos.

### 2.7. LDH Cytotoxicity Assay

The LDH assay was performed as described previously [35]. Briefly, after treatment with particles, control cells were treated with 1% (v/v) of Triton X-100 for 30 min to detect the maximum LDH release (positive control). The whole plates were then centrifuged at 1500 rpm for 5 min and 50 µL of the supernatant was transferred into a 96 well plate. 50 µL PBS and 100 µL of the LDH working solution, prepared according to the manufacturer’s instructions, were added per well and incubated at room temperature for 10 min. By the addition of 50 µL of 1 N HCl, the reaction was stopped. Absorbance was measured at 490 nm using a multi-well plate reader and analyzed by the software package SoftMaxPro (version 3.0, Molecular Devices, Ismaning, Germany). Cytotoxicity data are depicted as percentage relative to the positive control set to 100%.

### 2.8. AlamarBlue Viability Assay

The AlamarBlue^®^ assay was performed as described previously [35], using the same plate with exposed cells as for the LDH assay. The remaining medium was removed and 100 µL of diluted AlamarBlue^®^ reagent (1:20 in HBSS) was added. After 60 min in the incubator, the samples were analyzed using a multi-well plate fluorescent reader (Bio-Tek FL600, software package KC4 version 2.7, MWG-Biotech AG, Ebersberg, Germany) at 560 nm excitation and 620 nm emission wavelengths. Viability data are depicted as percentage relative to the negative control (untreated cells, 100%).

### 2.9. Protein Detection by Western Blot

Western blot was performed as described before [36]. The cells, which were seeded into 6 well plates, were treated with the particles according to the experimental design. After treatment, the cells were lysed in 2× Laemmli buffer (125 mM Tris–HCl, 4% SDS, 20% glycerol, 8% beta-mercaptoethanol, pH 6.8), boiled at 95 °C for 5 min, and sonicated in an ultrasonic water bath (Bandelin Sonorex, Berlin, Germany) for 10 min. Lysates were loaded on 12 or 15% gels depending on the molecular size of the protein to be detected for SDS-PAGE. After electrophoresis, the proteins were transferred onto Immobilon membranes (Millipore, Darmstadt, Germany). Membrane blocking was performed in 5% (w/v) non-fat dry milk powder in Tris-buffered saline containing 1% (v/v) Tween20 (TBS-T) for 1 h. Appropriate primary antibodies were applied in 5% (w/v) non-fat dry milk in TBS-T overnight at 4 °C. After incubation with appropriate HRP-conjugated secondary antibodies for 1 h, enhanced chemiluminescence (ECL) detection was performed according to the manufacturers’ instructions.

### 2.10. Statistical Analyses

Data are expressed as mean ± standard deviation (SD). The significance of difference between two mean values was assessed by the Student’s *t*-test or the Mann–Whitney Rank Sum test using the SigmaPlot software (version 11.0, Systat Software GmbH, Erkrath, Germany). A *p*-value < 0.05 was considered to be statistically significant.

## 3. Results and Discussion

### 3.1. Characterization of Silica Particles

In this study, we used commercial amorphous silica nanoparticles synthesized by different methods and of different sizes and surface modifications, as listed in Table 1. SiO_2_—12 nm, also known as Aerosil^®^ 200, is a hydrophilic pyrogenic silica NP produced by flame synthesis and was delivered as a powder. SiO_2_—70 nm NPs were produced by a wet process known as the Stöber method [37] and were delivered in an aqueous suspension. Amine- and carboxyl-modified silica NPs of the same size were used to study the effects of surface modification on the toxicity of colloidal silica NPs. Furthermore, the effect of size was studied by using 200 and 500 nm Stöber-synthesized silica particles. The primary particle diameter analyzed by TEM was used to calculate the specific surface area of the particles in m^2^/g. The particles were further characterized in medium without and with 10% FBS by dynamic light scattering (Table S1). In DMEM without FBS, all Stöber-synthesized particles showed a hydrodynamic diameter very close to their primary diameter, indicating a monodisperse suspension. The hydrodynamic diameter was slightly increased in the medium with 10% FBS, most likely due to the formation of a protein corona. SiO_2_—12 nm NPs were already aggregated to 232 nm in medium without FBS (presumably due to sintering [22]), and further agglomerated in medium with 10% FBS. A more detailed characterization of Aerosil^®^ 200 according to OECD test guidelines is provided in Mülhopt et al. [38]. In these studies, trace amounts of Ni (0.1 µg/g) and Cu (0.2 µg/g) were detected by inductively coupled plasma-mass spectrometry (ICP-MS), which, however, were below the level of toxicological relevance. 

### 3.2. Silica NPs Induced Apoptotic and Necrotic Cell Death Which Was Suppressed by the Presence of Serum

**Figure 1:**
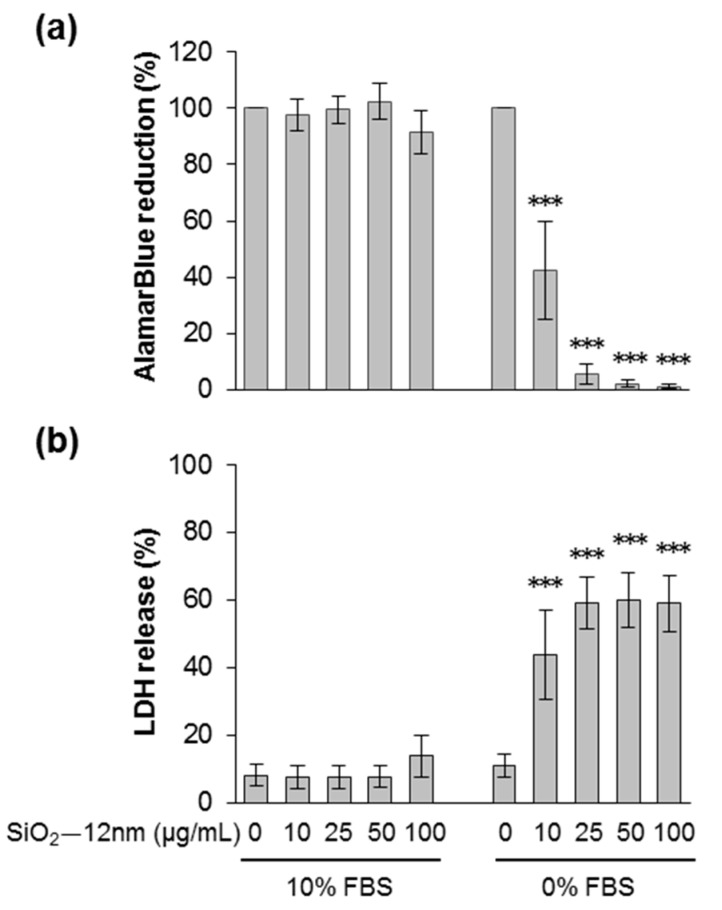
Cell viability decreased after incubation with silica NPs in the absence of fetal bovine serum (FBS). HCT116 wt cells were incubated with SiO_2_—12 nm NPs at the indicated concentration in the presence or absence of 10% FBS. After 24 h, cell viability was detected by the AlamarBlue assay (**a**). The values were normalized to the negative control (no particles were added, 100%). Lactate dehydrogenase (LDH) release is shown in (**b**). The values were normalized to the positive control (1% Triton X-100, 100%). The data represent the means of seven independent experiments ± SD performed with six replicates. *** *p* < 0.001 indicates significant differences in the response of cells treated with the same amount of NPs in the absence (0% FBS) or the presence of serum (10% FBS).

The effects of SiO_2_—12 nm NPs on cell viability after 24 h were tested using the AlamarBlue and the LDH release assays (Figure 1a,b). Viable cells reduce the AlamarBlue dye by mitochondrial dehydrogenases and thus change its color. Reduction of AlamarBlue did not change when cells were exposed to particles dispersed in medium with 10% FBS. However, it decreased dose-dependently after treatment in the absence of FBS, even at 10 µg/mL of SiO_2_—12 nm NPs. The results of the LDH assay correlate well with the AlamarBlue assay. Release of LDH is an indicator of plasma membrane damage. For cells treated with 10 µg/mL of SiO_2_—12 nm NPs in FBS-free medium, there was already a strong release of LDH, which further increased with higher doses. There was no LDH release detected when cells were exposed to particles suspended in medium with 10% FBS. The cytotoxic effects of SiO_2_—12 nm NPs observed after 48 h were similar to those after exposure for 24 h (Figure S1a,b), indicating a rapid onset of cell damage. Indeed, silica NPs triggered membrane rupture as early as 5 h after exposure (Figure S2a). In order to mimic the exposure of colon cells from the luminal side, we further reduced the amount of serum in the exposure medium. Interestingly, silica NPs also provoked membrane damage at 24 h in the presence of 1% FBS, albeit reduced compared to the levels induced in the total absence of serum (Figure S2b). The protective effects of just 1% FBS in the exposure medium were even more obvious at an earlier time point, i.e., 5 h after treatment (Figure S2a). In conclusion, silica NPs promoted cytotoxicity in HCT116 cells dependent on time, concentration, and the presence of serum, as previously reported for other cell types [15,16,20,22,23,39].

**Figure 2:**
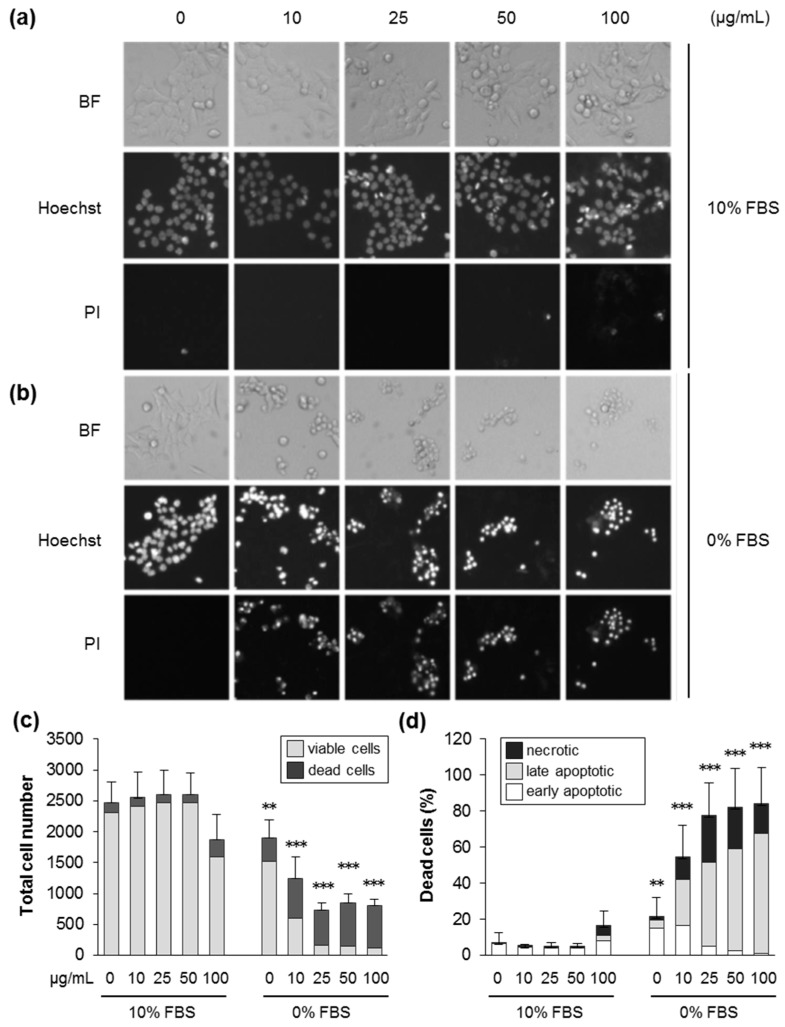
Silica NPs induce apoptotic and necrotic cell death in the absence of serum. HCT116 wt cells were incubated with SiO_2_—12 nm NPs at the indicated concentration in the presence (10% FBS) or absence (0% FBS) of serum. After 24 h, the cells were stained with Hoechst and propidium iodide (PI), and images were acquired by automated microscopy and analyzed by the scan^R software to deduce cell numbers and the different modes of cell death. (**a**,**b**) Representative images of cells treated as indicated in the brightfield, the Hoechst, and the PI channels. (**c**) The total cell number divided into living and dead cells after treatment, as indicated. (**d**) The percentage of dead cells relative to the total cell number divided into the different classes of cell death, as indicated. Data are represented as mean values of three independent experiments carried out with four replicates (*n* = 12). The error bars are SD values related to the total cell number (**c**) or the percentage of dead cells (**d**). ** *p* < 0.01, *** *p* < 0.001 indicate significant differences in the response of cells treated with particles at corresponding concentrations in the absence (0% FBS) or the presence of serum (10% FBS).

Next, we used automated fluorescence microscopy to further identify the mechanisms of cell death. After 24 h in the absence of FBS, the total cell number decreased significantly at increasing levels of SiO_2_—12 nm NPs, while, concomitantly, the proportion of dead cells increased (Figure 2c,d). Similar effects were found at 48 h of exposure (Figure S3). Dead cells showed typical features of cell death, such as DNA condensation and loss of membrane integrity, and were mainly categorized as late apoptotic and necrotic cells (Figure 2a,b,d, Video S1). As also documented above using the AlamarBlue and LDH assays (Figure 1), 10% serum in the exposure medium largely prevented cytotoxicity. In accordance with the rapid damage of the cell membrane detected by the LDH assay at 5 h in the absence of serum (Figure S2a), an early influx of propidium iodide indicative of membrane rupture was also observed at the single cell level (Figure S4a,b). Furthermore, in accordance with the LDH assay (Figure S2b), the presence of 1% FBS reduced cell death prompted by silica NPs (Figure S5a,b). These findings, together with previous results obtained with human colon carcinoma [15] and lung epithelial cells [24], indicate that the ratio of the specific particle surface area and concentration of proteins in the exposure medium has a critical impact on the occurrence of cell death upon treatment with silica NPs. 

### 3.3. Silica NPs Triggered Cell Death Dependent on Size, Specific Surface Area, and Surface Modification

**Figure 3:**
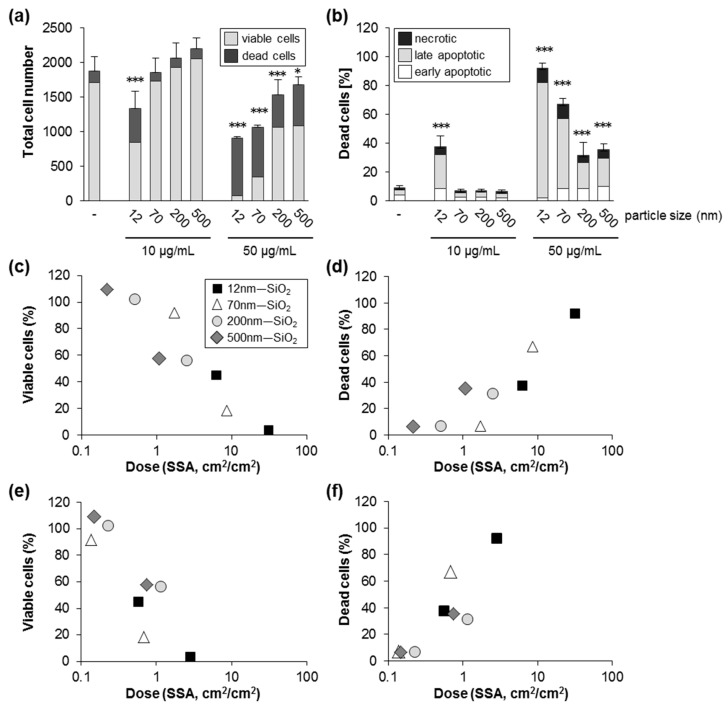
Silica NPs induce cell death dependent on size and specific surface area. HCT116 wt cells were incubated with particles as indicated in the absence of FBS. After 24 h, the cells were processed and analyzed as described in Figure 2. (**a**) The total cell number divided into living and dead cells. (**b**) The percentage of dead cells relative to the total cell number divided into the different classes of cell death as indicated. Data show mean values of two independent experiments carried out with four replicates (*n* = 8). The error bars are SD values related to the total cell number (**a**) or the percentage of total dead cells (**b**), respectively. * *p* < 0.05, *** *p* < 0.001 indicate significant differences in the response of cells treated with particles in comparison to untreated control cells (-). The percentage of viable (**c**) and dead cells (**d**) are plotted against the nominal specific particle surface area dose per cell area (cm^2^/cm^2^, logarithmic scale). For comparison (**e**,**f**), the calculated deposited dose has been used as a metric. Note that the nominal (**c**,**d**) and calculated (**e**,**f**) dose in case of larger particles (200 and 500 nm) were rather similar, whereas for nanoparticles (12 and 70 nm), only a small fraction was deposited. Further details are described under Methods.

To address the impact of size on silica NP induced cell death under serum-free conditions, we studied colloidal silica spheres with nominal diameters of 70, 200, and 500 nm (Table 1). Administration of particles with increasing diameter but at the same mass concentration (10 and 50 µg/mL) revealed an inverse relationship of particle size and cytotoxicity, i.e., larger particles were less toxic compared to smaller particles (Figure 3a). Similarly to the effects observed for SiO_2_—12 nm NPs derived from flame synthesis (Figure 2), colloidal silica NPs also provoked apoptotic and necrotic cell death dependent on dose and size (Figure 3b, Figure S6). Silica NPs are surface active materials and interact with biological surfaces, i.e., cell membranes. The specific particle surface area (SSA, cm^2^ at a defined mass or volume) increases linearly with a reciprocal decrease in particle size. The dose–response related to the nominal concentration in the exposure media indicated increased toxicity of smaller versus larger silica NPs (Figure 3a,b). However, normalization of the dose response curves to the administered SSA showed a clear correlation of SSA and cytotoxicity (Figure 3c). As the deposited, i.e., effective, dose of particles is controlled by sedimentation and diffusion, which depend on particle size and effective density, we also calculated the delivered cellular dose [33,40]. While for the larger particles of 200 and 500 nm there was no major difference in the effective SSA dose, for the smaller nanoparticles (12 and 70 nm), the effective SSA (Figure 3e,f) was drastically reduced compared to the nominal dose (Figure 3c,d). As deposition of nanoparticles is mainly driven by diffusion, the relative fraction of administered NPs interacting with cells was lower compared to the larger particles, highlighting the importance of computational modeling of the relative in vitro dose for hazard ranking of differently sized materials. Indeed, several in vitro and in vivo studies have shown that cytotoxicity of insoluble NPs better correlates with the SSA dose than with the particle mass dose [24,41,42,43]. 

**Figure 4:**
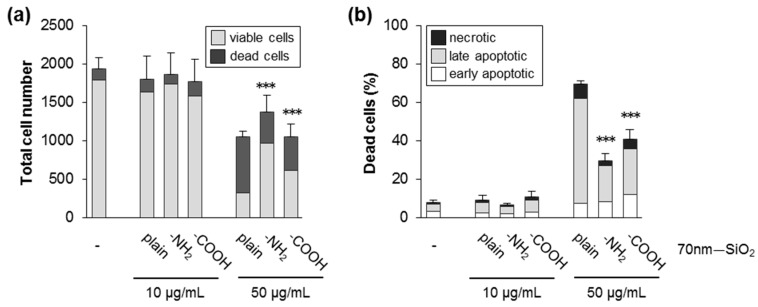
Surface modifications reduces cytotoxicity of silica NPs. HCT116 wt cells were exposed to SiO_2_—NPs (70 nm) with a plain, NH_2_—, or COOH—modified surface in the absence of FBS. After 24 h, the cells were processed and analyzed as described in Figure 2. (**a**) The total cell number divided into living and dead cells. (**b**) The percentage of dead cells relative to the total cell number divided into the different classes of cell death as indicated. Data show mean values of two independent experiments carried out with four replicates (*n* = 8). The error bars are SD values related to the total cell number (**a**) or the percentage of total dead cells (**b**), respectively. *** *p* < 0.001 indicates significant differences in the number of viable cells (**a**) and total dead cells (**b**) after administration of modified NPs compared to corresponding concentrations of plain particles.

Chemical surface functionalization of silica NPs modulates the cellular response [44,45]. Therefore, we also investigated in HCT116 cells the effects of colloidal silica NPs of the same size (70 nm) with a plain, NH_2_—, or COOH—modified surface. As observed in other cell models, chemical modification of the silica surface suppressed cytotoxicity (Figure 4a,b; Figure S7). Replacement of reactive silanol groups by functional groups is supposed to prevent their detrimental interaction with cellular membranes [46,47].

### 3.4. Silica NPs Promoted Cell Death Dependent on Caspases and Independent of p53 or BAX

**Figure 5:**
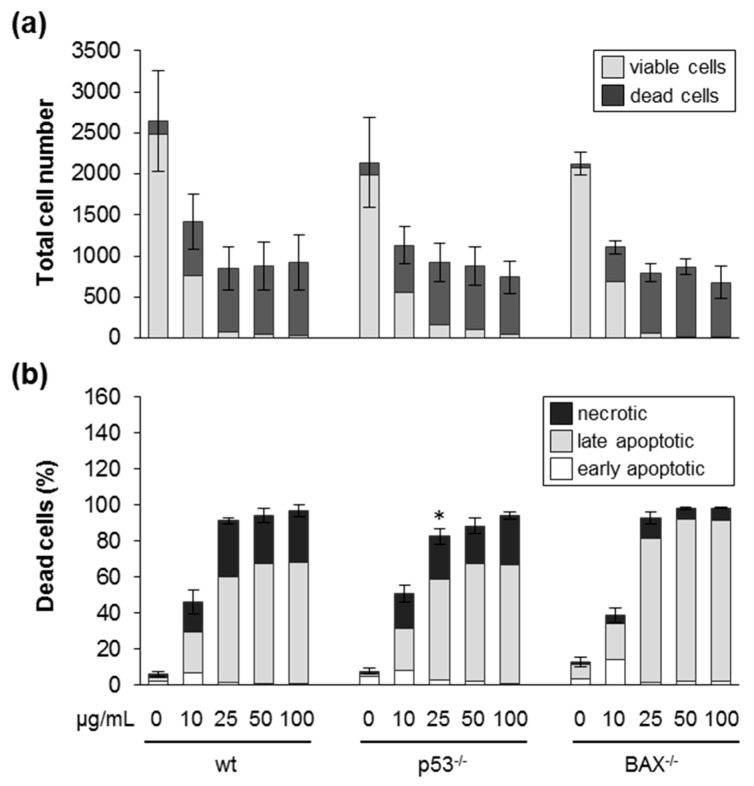
Silica NPs induce cell death independent of p53 and BAX. HCT116 wild-type (wt), p53 knock-out (^−/−^) and BAX knock-out (^−/−^) cells were incubated with SiO_2_—12 nm NPs as indicated in the absence of FBS. After 24 h, the cells were processed and analyzed as described in Figure 2. (**a**) The total cell number divided into living and dead cells. (**b**) The percentage of dead cells relative to the total cell number divided into the different classes of cell death as indicated. Data show mean values of five–six independent experiments with wt and p53^−/−^ cells. For BAX^−/−^ cells, one representative experiment out of two is shown. All experiments were carried out with three–four replicates. The error bars are SD values related to the total cell number (**a**) or the percentage of total dead cells (**b**), respectively. Note that there was no major difference in the response of p53^−/−^ or BAX^−/−^ compared to wild-type cells. * *p* < 0.05.

Silica NPs induced cell death in HCT116 cells via apoptosis and necrosis, as evidenced by the image analysis outlined above. Execution of cell death is controlled by intricate signaling networks and is of relevance for the toxicity of several nanomaterials [48]. The most prominent determinants of cell death are the tumor suppressor protein p53 and its target BAX [49]. p53 is a transcription factor which is activated due to cellular stress, and regulates cell growth, cell cycle progression, and cell death via apoptosis, necroptosis, or ferroptosis. BAX acts on mitochondria to promote apoptosis and is regulated by p53, which upregulates transcription of the gene encoding BAX. In order to further investigate the mechanism of cell death triggered by silica NPs, we explored wild-type HCT116 cells and their isogenic derivatives in which the p53 gene was deleted by homologous recombination [28]. Compared to wild-type cells, silica-NP-induced cell death monitored by automated microscopy at 24 h was not reduced in p53 knock-out cells (Figure 5a,b). The similar sensitivity of p53 deficient cells was also confirmed by the LDH and AlamarBlue assays (compare Figure S8a,b and Figure 1). Additionally, at an earlier time point (5 h), no reduced cell death but rather, enhanced cytotoxicity could be observed in p53 knock-out cells (Figure S9a,b; Figure S10; Figure S2a). Similarly, at 48 h after exposure to nanosilica, no major difference in the percentage of cell death was obvious when wildtype and p53 knock-out cells were compared (Figure S11). Next, we analyzed the involvement of the p53 target BAX in the execution of nanosilica-induced cell death. Again, as found in the case of p53 deficient cells, BAX knock-out cells were not protected against the detrimental action of nanosilica (Figure 5a,b). In summary and in contrast to the action of some genotoxins [28,34], the p53–BAX axis seems not to be involved in the execution of cell death prompted by exposure to nanosilica in HCT116 cells. Previous reports have suggested a pro-apoptotic role of p53 and BAX in nanosilica-induced cell death [50,51]. After treatment with silica NPs, an upregulation of the p53 and BAX proteins in human fetal hepatocytes and hepatoma cells could be demonstrated, which correlates with the onset of cell death. However, further studies in these and other cell types are warranted to really confirm a functional role of p53 and BAX (e.g., by genetic or pharmacological interference) in nanosilica-induced cell death. 

**Figure 6:**
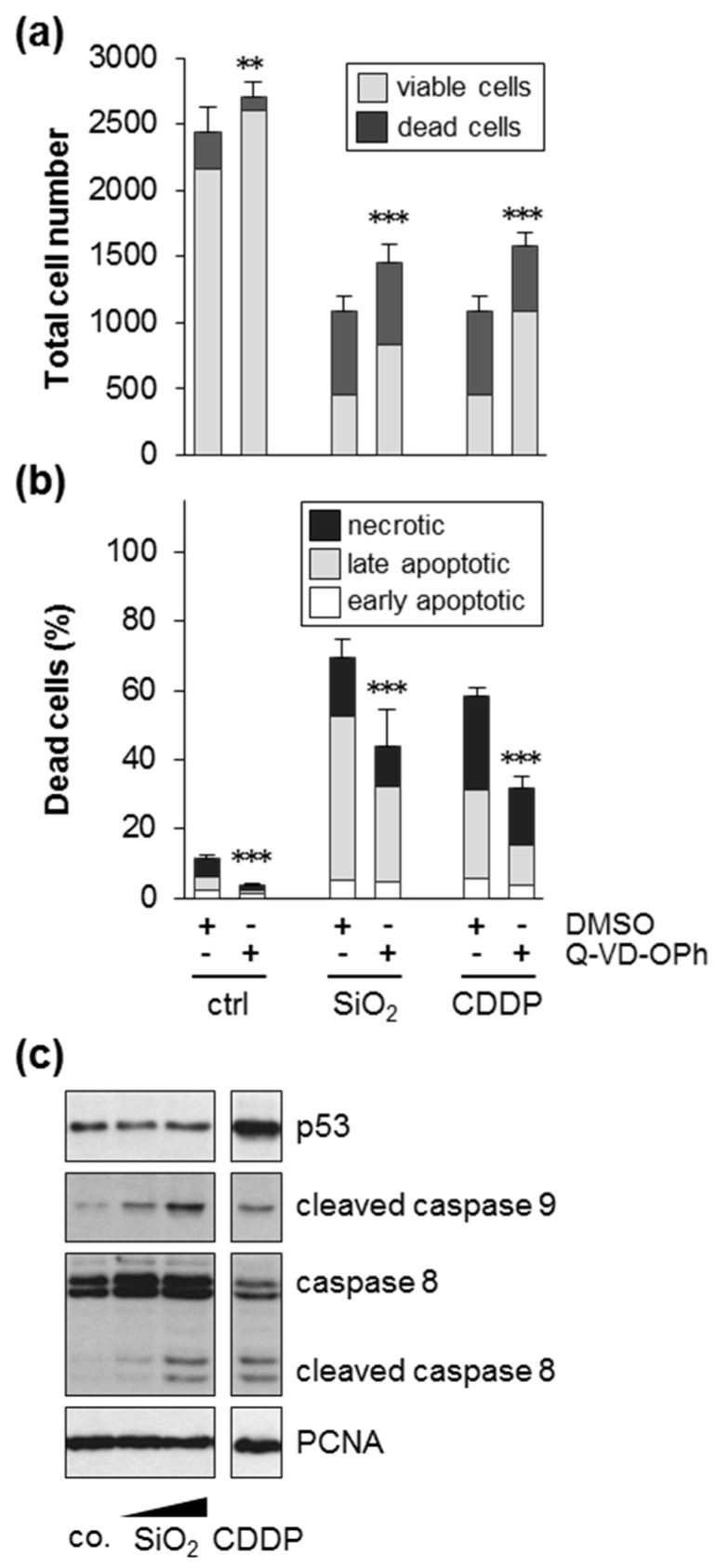
Silica NPs induce cell death dependent on caspases. HCT116 wt cells were incubated with 10 µg/mL SiO_2_—12 nm NPs or with 50 µM of the positive control compound cisplatin (CDDP) in medium without FBS. Samples were co-treated as indicated either with the pan-caspase inhibitor Q-VD-OPh (10 µM) dissolved in DMSO (0.5%) or simply the solvent (DMSO, 0.5%). After 24 h, the cells were processed and analyzed as described in Figure 2. (**a**) The total cell number divided into living and dead cells; (**b**) the percentage of dead cells relative to the total cell number divided into the different classes of cell death as indicated. Data show mean values of two independent experiments carried out with four replicates (*n* = 8). The error bars are SD values related to the total cell number (**a**) or the percentage of total dead cells (**b**), respectively. *** *p* < 0.001 indicates significant differences in the response of cells treated with or without the pan-caspase inhibitor. (**c**) Activation, i.e., cleavage of caspase 8 and 9 was analyzed by western blotting after treatment with 0 (co.), 5 and 10 µg/mL SiO_2_—12 nm NPs or with 50 µM CDDP in the absence of serum for 22 h. PCNA was used as a loading control. The results are representative of two independent experiments.

Finally, we addressed the involvement of caspases in silica-NP-induced cell death. Indeed, inhibition of caspases partially protected cells from silica-NP-induced cell death, evidenced by an increase of viable cells (Figure 6a) and a decrease in apoptotic and necrotic cells (Figure 6b). As also shown for the positive controls, which were treated with the genotoxin cisplatin, caspases are required for the promotion of cell death initiated by silica NPs. Next, we monitored cleavage of caspase 8 and caspase 9, which act as initiator caspases in the extrinsic (as part of the death-inducing signaling complex) and intrinsic (as part of the apoptosome) apoptotic pathways, respectively [52]. Exposure of cells to increased amounts of silica NPs triggered cleavage of caspase 8 and 9 (Figure 6c), in line with a critical role of caspase activity in the execution of cell death (Figure 6a,b). Furthermore, in contrast to the positive control cisplatin, silica NPs did not elevate the protein levels of p53 (Figure 6c). This also supports the loss of function experiments, i.e., similar cytotoxicity in wt and p53 knock-out cells after exposure to silica NPs (Figure 5a,b).

## 4. Conclusions and Perspectives

Engineered silica nanoparticles are ingested as food additives and belong, together with titania NPs, to the most frequently used nanomaterials in food products [8]. The present study revealed cytotoxic actions of silica NPs in the colon cancer cell line HCT116, specifically in the absence or presence of low amount of serum, corroborating previous studies in the two different human colon cancer cell lines Caco-2 and HT29 [13,14]. In Caco-2 cells, a pro-inflammatory action of silica NPs was also observed indicated by the release of IL-8 [53]. Interestingly, differentiated Caco-2 cells were less sensitive to the noxious effects of silica NPs. The physiological relevance of in vitro studies needs to be considered, and specific test systems and guidelines still need to be optimized in case of hazard and risk assessment of NPs upon ingestion [8]. With respect to dosimetry, we chose a realistic dose range for all different silica nanoparticles below the calculated relevant in vitro dose of about 2 µg/cm^2^ (NP mass/cellular surface area), approximated from the daily average intake of food grade silica (1.8 mg/body weight/day) in humans [8]. Unfortunately, there is a lack of in vivo studies investigating toxicity of silica NPs in the gut. Recently, production of pro-inflammatory cytokines upon oral administration of nanosilica (2.5 mg/kg bw/day) for 7 days was observed in mice, which coincided with slight histological changes, i.e., crypt damage and inflammatory cell infiltration [10]. Thus, it is tempting to speculate that microlesions in the gut epithelium due to cell death could contribute to the inflammatory symptoms. In the colonic crypts, there is a constant renewal of epithelial cells, which originate from stem cells and progressively differentiate to become mature enterocytes. Clearly, more detailed studies in vivo are warranted to address the impact of nanosilica on the viability of epithelial cells, and also in relation to their differentiation status. It is of note that the in vitro experiments of Wiemann et al. with alveolar macrophages cultivated under serum-free conditions have been demonstrated to predict the short-term inhalation toxicity in vivo for 18 different nanomaterials, including nanosilica [54]. Upon inhalation, NPs are covered by the lung lining fluid, mostly comprised of surfactant (primarily phospholipids) and a small amount of proteins, and are not covered by serum proteins. Therefore, in vitro exposure of lung cells in the presence of high levels of serum proteins (i.e., 10% FBS) does not closely mimic the physiological conditions in the lung. Concerning a realistic exposure scenario for the gastrointestinal tract, NPs are embedded in a complex food matrix, which is further altered upon ingestion and digestion. Therefore, the corona, i.e., the adsorbed molecules on the NP surface, is ill-defined and comprised of a multitude of different molecules including carbohydrates, protein, and lipids. However, compared to localization of NPs in the blood stream after direct injection (in the case of medical applications) or after translocation from the gut into the portal vein, the amount of serum proteins in the gut lumen is much lower. Hence, the presence of high levels of serum proteins as used in most in vitro experiments might passivate the silica surface and suppress the interaction of silica NPs with critical targets such as the cellular membrane or certain receptors. Although in vitro experiments employing monocultures can be used to address specific processes and pathways, co-cultures of colon epithelial cells together with mucus producing goblet cells or lymphoid cells might even be better suited to predicting the more complex situation in vivo. Indeed, exposure of differentiated Caco-2 cells co-cultured with mucus producing HT29-MTX cells to titania NPs triggered the production of reactive oxygen species [55,56,57] and an inflammatory response, which was absent in Caco-2 cells grown as monocultures.

Finally, in vitro studies in toxicology often rely on the use of transformed cancer cells, as primary cells of various organs are difficult to cultivate and are often not available. Therefore, adverse effects observed in vitro might indicate toxicity in vivo, but need to be confirmed in follow-up investigations. However, the unintended toxicity of NPs determined in vitro might also be beneficial and exploited to selectively eliminate cancer cells. Specifically, silica NPs also kill p53-deficient colon cancer cells, as shown here, which are resistant to chemotherapy by conventional drugs such as cisplatin. Hence, oral delivery of silica NPs could be envisioned for the treatment of colon cancer. Selective accumulation of silica NPs via tumor-targeting ligands could be combined with the tunable unmasking of the silica surface, e.g., by dePEGylation initiated by near infrared radiation, as shown recently [58].

## Figures and Tables

**Table 1:** Characteristics of silica nano- and microparticles.

Particles	Surface Modification	Nominal Primary Particle Diameter (nm) ^a^	Primary Particle Diameter (nm) ^b^	Specific Surface Area (m^2^/g)
SiO_2_—12 nm (Aerosil^®^ 200)	plain	12	15 ± 10 ^d^	200 ± 25 ^a^
SiO_2_—70 nm	plain	70	55 ± 7 ^e^	55 ^c^
SiO_2_-NH_2_—70 nm	–NH_2_	70	55 ± 7 ^e^	55 ^c^
SiO_2_-COOH—70 nm	–COOH	70	64 ± 7	47 ^c^
SiO_2_—200 nm	plain	200	190 ± 20	16 ^c^
SiO_2_—500 nm	plain	500	433 ± 25	6.9 ^c^

^a^ Data provided by the supplier, ^b^ analyzed by transmission electron microscopy (TEM), ^c^ calculated from the primary particle size, and the density 2.0 g/cm^3^ for SiO_2_ nanoparticles (NPs). ^d^ Already published in Reference [38], ^e^ already published in Reference [25].

## Supplementary Materials

The following are available online at https://www.mdpi.com/2079-4991/9/8/1172/s1, Table S1: Characterization of SiO_2_ particle suspensions. Table S2: Nominal and effective i.e., calculated dose of differently sized SiO_2_ particles. Figure S1: Cell viability decreases after incubation with SiO_2_—12 nm NPs for 48 h in the absence of FBS. Figure S2: Low amount of serum (1% FBS) delays and reduces but does not totally prevent membrane damage provoked by SiO_2_—12 nm NPs. Figure S3: Cell death upon treatment with SiO_2_—12 nm NPs for 48 h in the absence of FBS. Figure S4: SiO_2_—12 nm NPs disturb the integrity of the cell membrane in the absence of FBS as indicated by the influx of propidium iodide already after 5 h. Figure S5: The toxic effects of SiO_2_—12 nm NPs after 24 h are diminished in the presence of 1% FBS. Figure S6: Silica NPs induce apoptotic and necrotic cell death dependent on size. Figure S7: Cytotoxicity of silica NPs is suppressed by surface modification. Figure S8: Cell viability decreases after incubation of HCT p53^−/−^ cells with SiO_2_—12 nm NPs for 24 h in the absence of FBS. Figure S9: HCT116 p53^−/−^ cells are more sensitive to SiO_2_—12 nm NPs than HCT116 wt cells. Figure S10: Low amount of serum (1% FBS) reduces membrane damage provoked by SiO_2_—12 nm NPs. Figure S11: The response of HCT116 wt and p53^−/−^ cells to SiO_2_—12 nm NP exposure after 48 h is similar. Figure S12: Summary of the results from the different toxicity assays to demonstrate the similar sensitivity of HCT wt and p53^−/−^ cells to SiO_2_—12 nm NPs dependent on the concentration of FBS. Video S1: Time course of cell death after incubation to SiO_2_—12 nm NPs in the absence of serum.
