# Supplementary material for: Silica Nanoparticles Provoke Cell Death Independent of p53 and BAX in Human Colon Cancer Cells
Source: Nanomaterials (Basel). 2019 Aug 16;9(8):1172. doi: 10.3390/nano9081172 (PMC6724124; doi:10.3390/nano9081172)
Supplement: Supplementary file 1 [file nanomaterials-09-01172-s001.zip › nanomaterials-558679 supplementary .pdf]

Article

Silica Nanoparticles Provoke Cell Death Independent of p53 and BAX in Human Colon Cancer Cells

Susanne Fritsch-Decker, Zhen An, Jin Yan, Iris Hansjosten, Marco Al-Rawi, Ravindra Peravali, Silvia Diabaté * and Carsten Weiss *

Institute of Toxicology and Genetics, Karlsruhe Institute of Technology, Hermann-von-Helmholtz-Platz 1, 76344 Eggenstein-Leopoldshafen, Germany

* Correspondence: silvia.diabate@kit.edu (S.D.); carsten.weiss@kit.edu (C.W.); Tel.: +49-72160822692 (S.D.); +49-72160824906 (C.W.)

Received: 10 July 2019; Accepted: 12 August 2019; Published: date

Table S1. Characterization of SiO_2 particle suspensions. The particles were directly suspended in DMEM in the presence (10% FBS) or absence (0% FBS) of serum. The hydrodynamic diameters (d_H) were analyzed at a particle concentration of 50 - 100 µg/mL. Data are means ± SD of 3 - 5 measurements. .

Particles	Hydrodynamic diameter in DMEM with	
	0% FBS	10% FBS
SiO_2—12nm[a]	232 ± 8	2245 ± 637
SiO_2—70nm [b]	51 ± 0.5	103 ± 2
SiO_2-NH_2—70nm [b]	76 ± 35	113 ± 47
SiO_2-COOH—70nm	89 ± 33	125 ± 60
SiO_2—200nm	232 ± 56	268 ± 98
SiO_2—500nm	521 ± 123	549 ± 239

[a]already published in (Leibe et al., 2019), [b]already published in (Hsiao et al., 2019).

Table S2. Nominal and effective i.e. calculated dose of differently sized SiO_2 particles. The cells were incubated with 50 µg/mL particles in DMEM without FBS for 24 h.

**Particles**	**Nominal mass dose** [a] (µg/cm²)	**SSA of deposited NPs (nominal)** [b] (cm²/cm²)	**Effective density** [c] (g/cm³)	**RID**[d] **(effective dose)** (µg/cm²)	**SSA** [e] **of deposited NP (effective dose)** (cm²/cm²)
SiO_2—12 nm (Aerosil®200)	15.6	31.3	1.11	1.41	2.83
SiO_2—70 nm	15.6	8.59	1.36	1.25	0.69
SiO_2—200 nm	15.6	2.50	1.57	7.06	1.13
SiO_2—500 nm	15.6	1.08	1.52	10.77	0.74

[a] Assuming complete deposition. [b] Deposited specific surface area (SSA) calculated from the nominal mass dose and data in Table 1. [c] Measured by VCM (DeLoid et al., 2014) assuming no solubility of the particles (n=3). [d] Relative In Vitro Dose (RID) calculated by the distorted grid (DG) nanotransport simulator (DeLoid et al., 2015) assuming "sticky" conditions. [e] Deposited specific surface area (SSA) calculated from RID and data in Table 1.

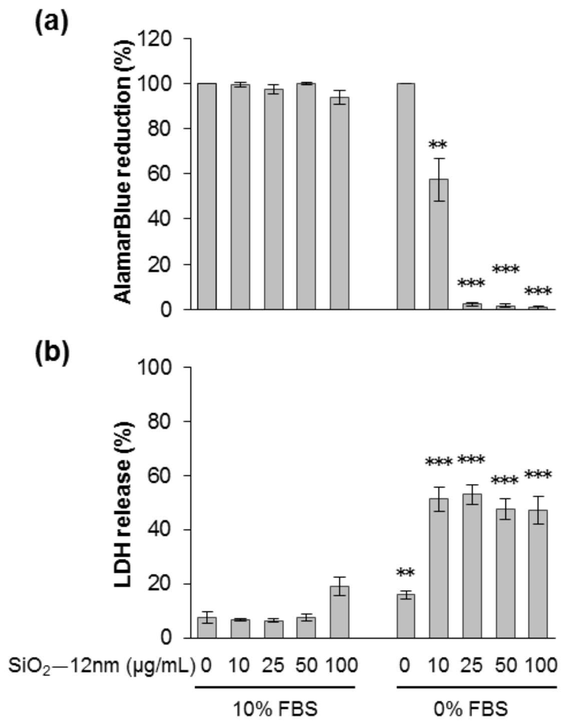

Figure S1. Cell viability decreases after incubation with SiO_2—12nm NPs for 48h in the absence of FBS. HCT116 wt cells were incubated with SiO_2—12nm NPs at the indicated concentration in the presence or absence of 10% FBS. After 48h, cell viability was detected by the AlamarBlue assay **(a)**. The values were normalized to the negative control (no particles were added, 100%). LDH release is shown in **(b)**. The values were normalized to the positive control (1% Triton X-100, 100%). The data represent means of three independent experiments ± SD performed with 6 replicates. **p < 0.01, ***p < 0.001 indicates significant differences in the response of cells treated with the same amount of NPs in the absence (0% FBS) or the presence of serum (10% FBS).

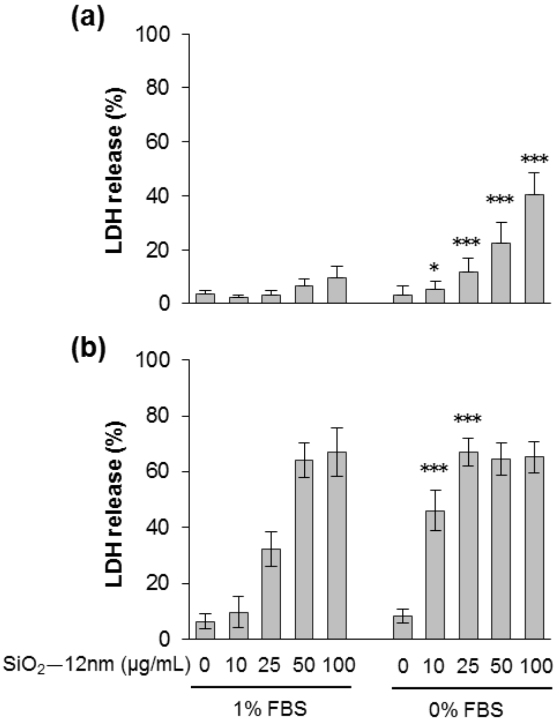

Figure S2. Low amount of serum (1% FBS) delays and reduces but does not totally prevent membrane damage provoked by SiO_2—12nm NPs. HCT116 wt cells were incubated with SiO_2—12nm NPs at the indicated concentration in the presence or absence of 1% FBS. After 5h **(a)** and 24h **(b)**, cytotoxicity was detected by the LDH assay. The values were normalized to the positive control (1% Triton X-100, 100%). The data represent means of four **(a)** or three **(b)** independent experiments ± SD performed with 4 replicates. *$p < 0.05$, ***$p < 0.001$ indicates significant differences in the response of cells treated with particles at corresponding concentrations in the absence (0% FBS) or the presence of reduced amount of serum (1% FBS).

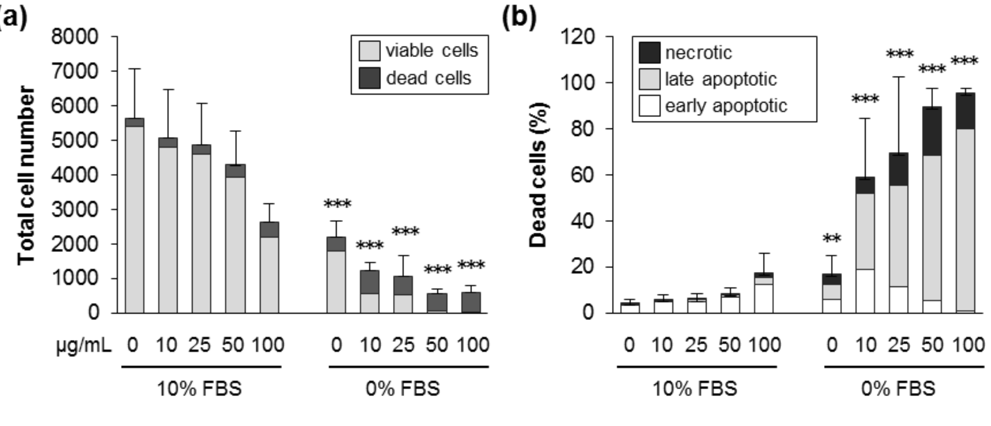

Figure S3. Cell death upon treatment with SiO_2—12nm NPs for 48 h in the absence of FBS. HCT116 wt cells were incubated with SiO_2—12nm NPs at the indicated concentration in the presence (10% FBS) or in the absence (0% FBS) of serum. After 48 h, the cells were processed and analysed as described in Figure 2. **(a)** shows the total cell number divided into living and dead cells after treatment as indicated. **(b)** shows the percentage of dead cells relative to the total cell number divided into the different classes of cell death. Data show mean values of two experiments carried out with four replicates (n=8). The error bars are SD values related to the total cell number **(a)** or the percentage of total dead cells **(b)**. **$p < 0.01$, ***$p < 0.001$ indicates significant differences in the response of cells treated with the same amount of NPs in the absence (0% FBS) or the presence of serum (10% FBS).

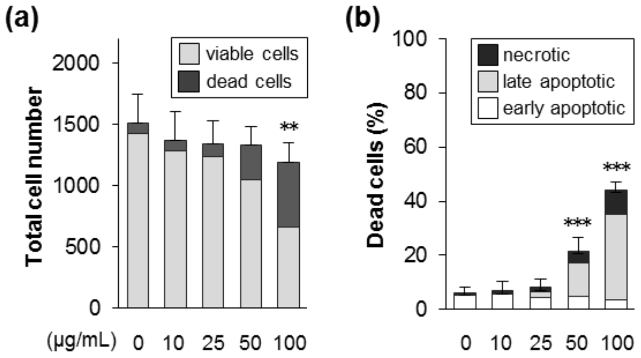

Figure S4. SiO_2—12nm NPs disturb the integrity of the cell membrane in the absence of FBS as indicated by the influx of propidium iodide already after 5 h. HCT116 wt cells were incubated with SiO_2—12nm NPs at the indicated concentration in the absence of FBS. After 5 h, the cells were processed and analysed as described in Figure 2. **(a)** shows the total cell number divided into living and dead cells. **(b)** shows the percentage of dead cells relative to the total cell number divided into the different classes of cell death as indicated. Data show mean values of 2 experiments carried out with four replicates (*n*=8). The error bars are SD values related to the total cell number **(a)** or the percentage of total dead cells **(b)**. **p < 0.01 and ***p < 0.001 indicates significant differences in the response of cells treated with particles compared to untreated control.

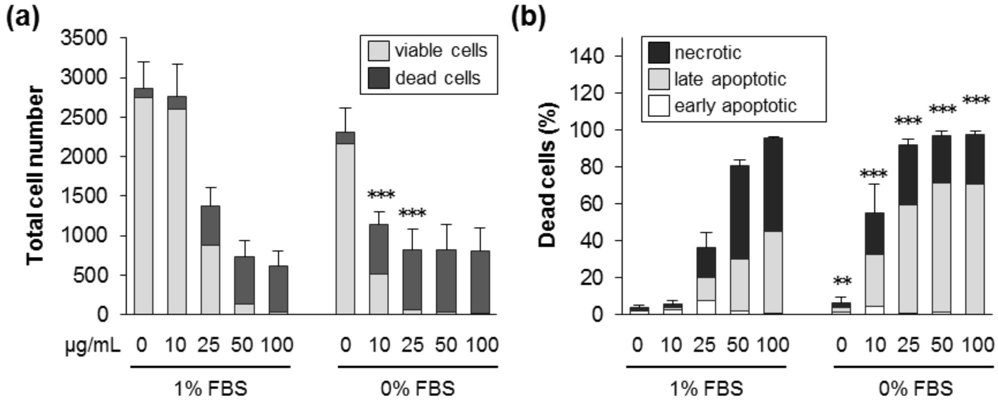

Figure S5. The toxic effects of SiO_2—12nm NPs after 24h are diminished in the presence of 1% FBS. The cells were treated, processed and analyzed as described in the legend of Fig. 2. Data show mean values of 4 experiments carried out with four replicates (n=16). The error bars are SD values related to the total cell number **(a)** or the percentage of total dead cells **(b)**. ** p < 0.01, ***p < 0.001 indicates significant differences in the response of cells treated with particles at corresponding concentrations in the absence (0% FBS) or the presence of reduced amount of serum (1% FBS).

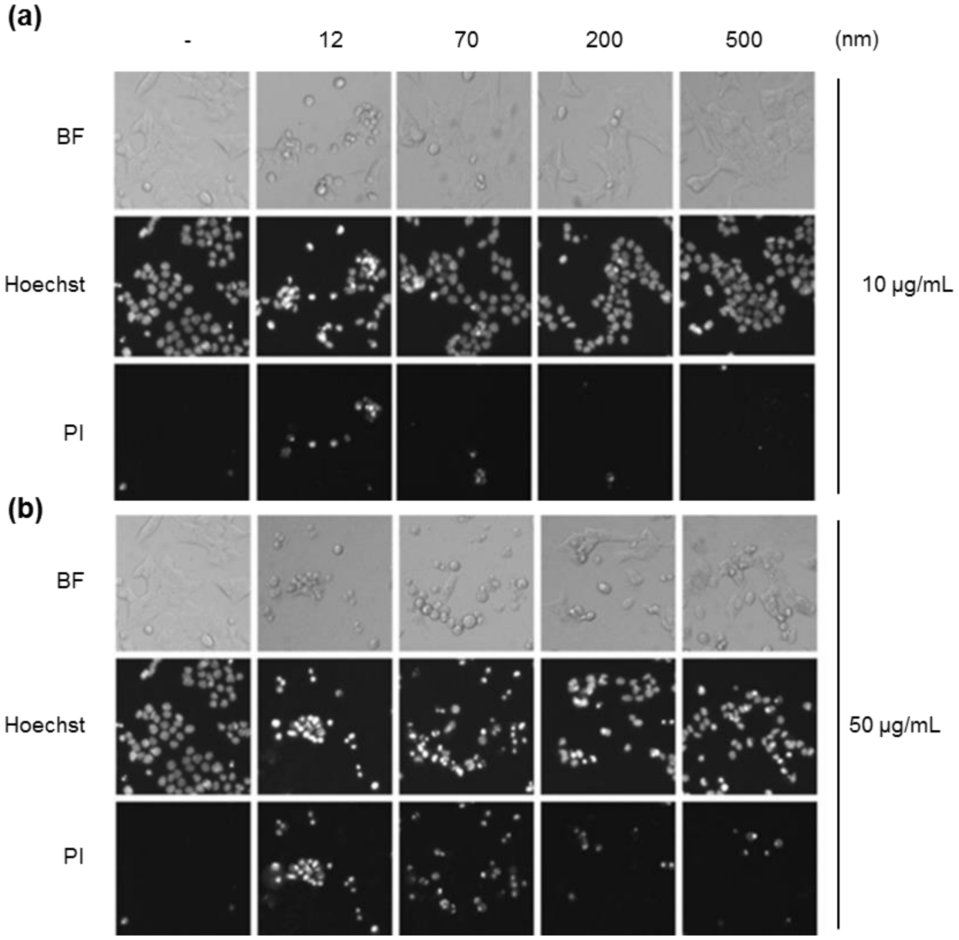

Figure S6. Silica NPs induce apoptotic and necrotic cell death dependent on size. HCT116 wt cells were incubated with 0 (-), 10 and 50 µg/mL SiO_2—particles of the indicated size in the absence of FBS. After 24 h, the cells were stained with Hoechst and PI. Representative images in the brightfield, the Hoechst and the PI channel of cells treated with 10 **(a)** and 50 µg/mL **(b)** of particles, respectively, are shown.

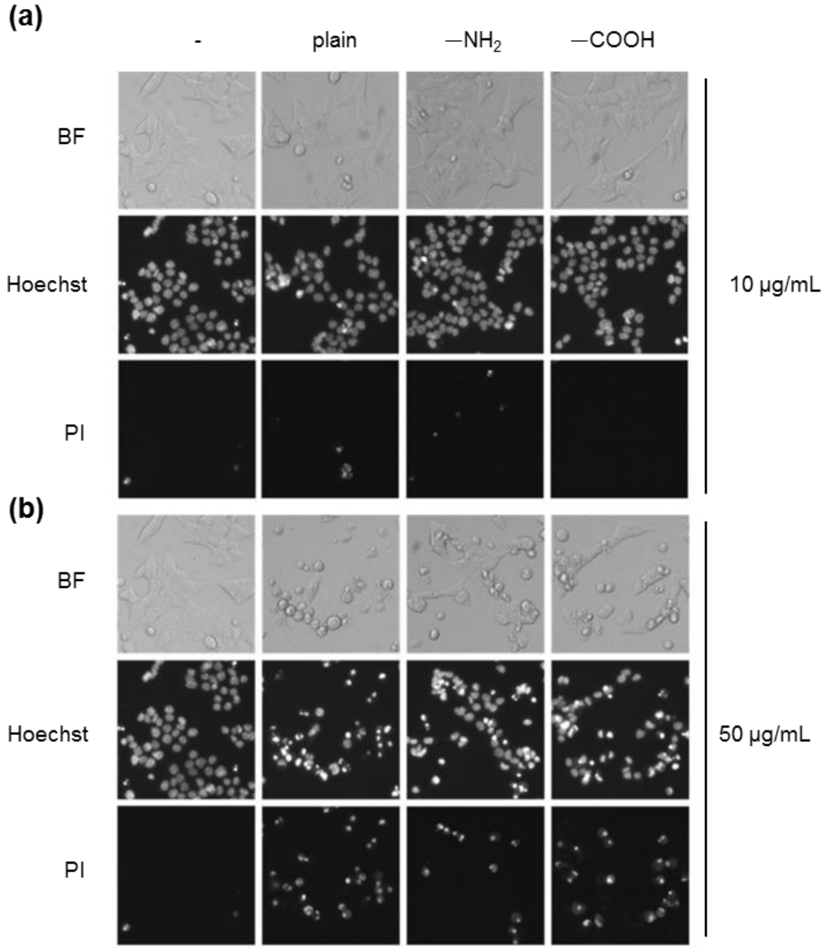

Figure S7. Cytotoxicity of silica NPs is suppressed by surface modification. HCT116 wt cells were incubated with 0 (-), 10 and 50 µg/mL SiO_2—70nm-plain or SiO_2—70nm-NH_2 or SiO_2—70nm-COOH NPs in the absence of FBS. After 24 h, the cells were stained with Hoechst and PI and analyzed by automated microscopy. Representative images in the brightfield, the Hoechst and the PI channel of cells treated with 10 **(a)** and 50 µg/mL **(b)** SiO_2 NPs, respectively, are shown.

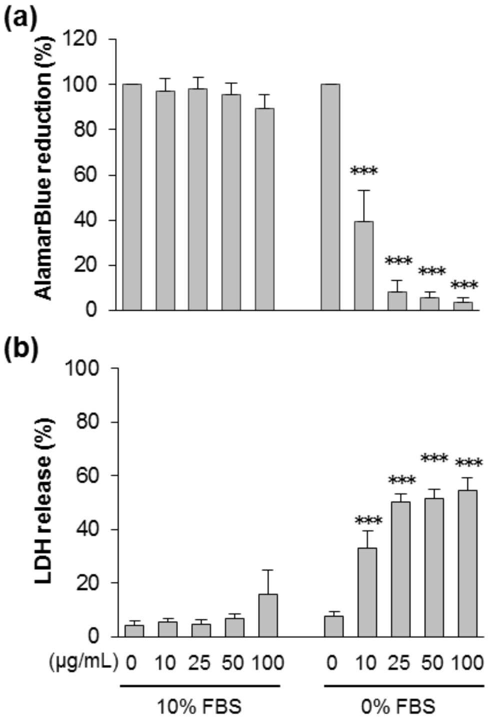

Figure S8. Cell viability decreases after incubation of HCT p53$^{-/-}$ cells with SiO_2—12nm NPs for 24h in the absence of FBS (0% FBS). The cells were incubated with SiO_2—12nm NPs at the indicated concentration in the presence or absence of 10% FBS. After 24 h, cell viability was detected by the AlamarBlue assay **(a)**. The values were normalized to the negative control (no particles were added, 100%). LDH release is shown in **(b)**. The values were normalized to the positive control (1 % Triton X-100, 100%). The data represent means of four independent experiments ± SD performed with 4 replicates. ***p < 0.001 indicates significant differences in the response of cells treated with the same amount of NPs in the absence (0% FBS) or the presence of serum (10% FBS).

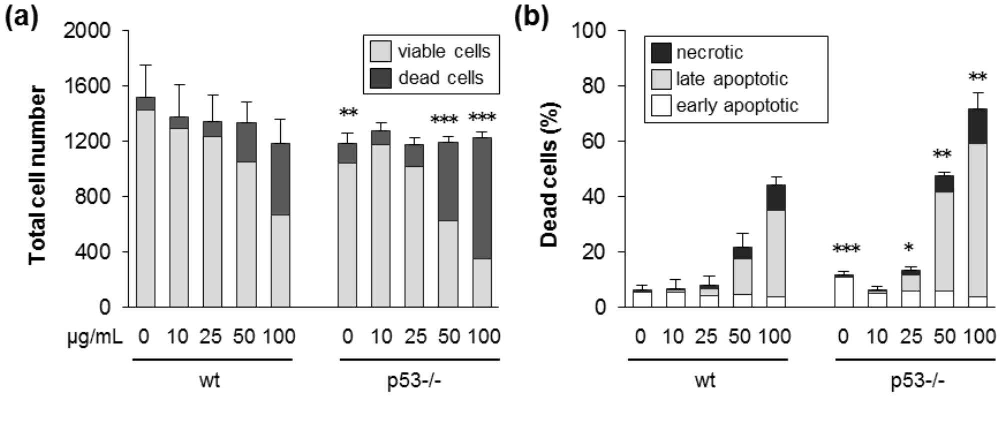

Figure S9. HCT116 p53$^{-/-}$ cells are more sensitive to SiO_2—12nm NPs than HCT116 wt cells. The cells were incubated with SiO_2—12nm NPs at the indicated concentration in the absence of FBS (0% FBS). After 5 h, the cells were processed and analysed as described in Figure 2. **(a)** shows the total cell number divided into living and dead cells after treatment as indicated. **(b)** shows the percentage of dead cells relative to the total cell number divided into the different classes of cell death. Data show mean values of two experiments with wt cells and one experiment wit p53$^{-/-}$ cells each carried out with four replicates (*n*=8; *n*=4). The error bars are SD values related to the total cell number **(a)** or the percentage of total dead cells **(b)**. *p < 0.05, **p < 0.01, ***p < 0.001 indicates significant differences in

the response of wt and p53$^{-/-}$ cells treated with the same amount of NPs assessed for either (a) the number of viable cells or (b) the percentage of dead cells.

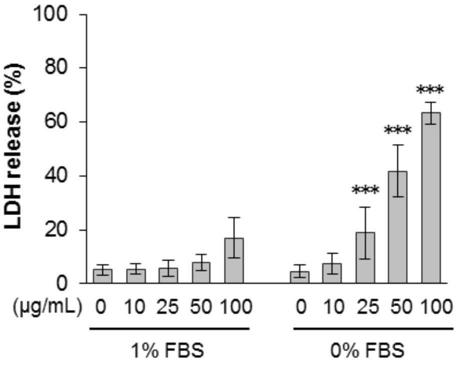

Figure S10. Low amount of serum (1% FBS) reduces membrane damage provoked by SiO_2—12nm NPs. HCT116 p53$^{-/-}$ cells were incubated with SiO_2—12nm NPs at the indicated concentration in the presence or absence of 1% FBS. After 5h, cytotoxicity was detected by the LDH assay. The values were normalized to the positive control (1% Triton X-100, 100%). The data represent means of four independent experiments ± SD performed with 4 replicates. ***$p < 0.001$ indicates significant differences in the response of cells treated with particles at corresponding concentrations in the absence (0% FBS) or the presence of reduced amount of serum (1% FBS).

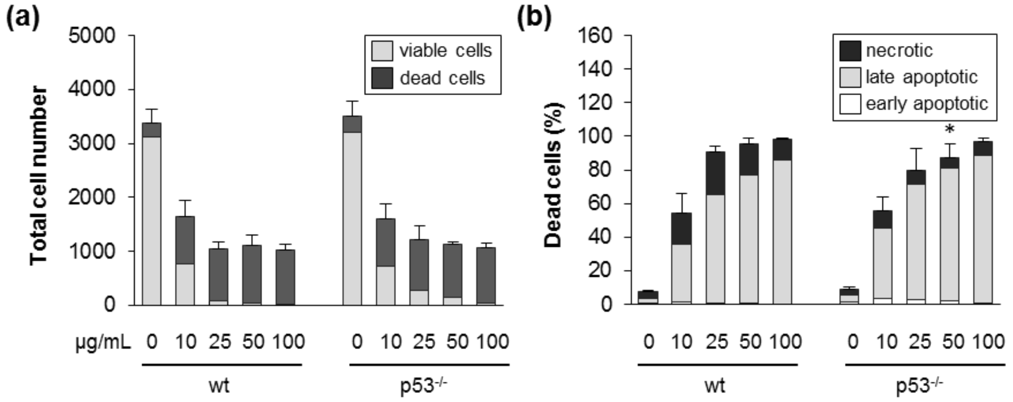

Figure S11. The response of HCT116 wt and p53$^{-/-}$ cells to SiO_2—12nm NP exposure after 48 h is similar. The cells were incubated with SiO_2—12nm NPs at the indicated concentrations in the absence of FBS (0% FBS). After 48 h, the cells were processed and analysed as described in Figure 2. **(a)** shows the total cell number divided into living and dead cells after treatment as indicated. **(b)** shows the percentage of dead cells relative to the total cell number divided into the different classes of cell death. Data show mean values of two experiments each carried out with four replicates (n=8). The error bars are SD values related to the viable cell number **(a)** or the percentage of total dead cells **(b)**. *$p < 0.05$.

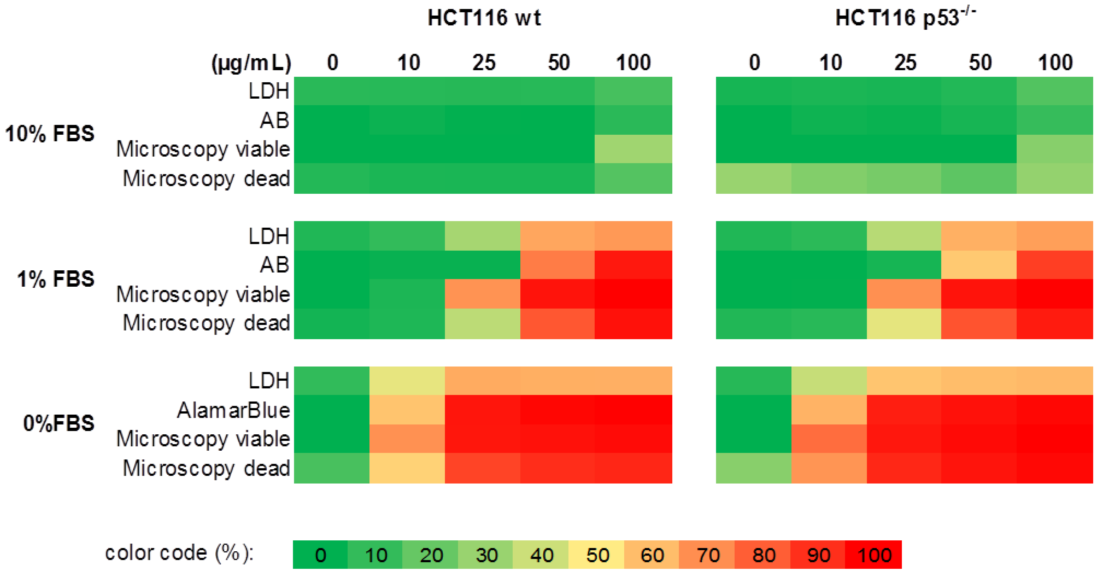

Figure S12. Summary of the results from the different toxicity assays to demonstrate the similar sensitivity of HCT wt and p53$^{-/-}$ cells to SiO_2—12nm NPs dependent on the concentration of FBS. The data of the LDH assay represent the percentage of dead cells relative to the Triton control. For the AlamarBlue (AB) assay, the relative difference (%) in AB reduction of treated cells relative to the medium control is plotted. In case of the microscopy assay, the the relative difference (%) in the number of viable cells (Microscopy viable) and increased percentage of dead cells (Microscopy dead) were related to the medium control which is set to 100%. All data are derived from 24 h exposure experiments. The color code represents the percentage of affected cells from 0% (green) to 100 % (red).

Videos

Video S1. Time course of cell death after incubation to SiO_2—12nm NPs in the absence of serum.

In order to analyze HCT116 cells by real-time imaging at the single cell level, cells were first seeded in 96-well plates and incubated overnight. On the next day, cells were directly incubated with 0.1 µg/mL Hoechst and 0.083 µg/mL PI, respectively, for 1 h at 37 °C and 5% CO_2. Then, cells were incubated in the absence of serum (0% FBS) with 25 µg/mL SiO_2—12 nm NPs over 24 h in a microscope incubator box as described under Methods. Every 15 min **(a)** and 10 min **(b)**, two images per well and channel (bright-field, Hoechst and PI, respectively) were acquired. Images were converted to movies using the NIH ImageJ Software. For comparison, cells grown in the presence (10% FBS) or absence (0% FBS) of serum without additional particle exposure are shown. In **(a)** merged images from all channels (bright-field, Hoechst and PI) are depicted. Note that cells proliferate undisturbed in the presence of serum (left movie) and some cell death occurred in cells cultivated in the absence of serum (middle movie). However, upon treatment with silica NPs a rapid increase in apoptotic and necrotic cells can be observed as evidenced by increased Hoechst and PI staining, respectively. Also in the bright-field channel typical features of programmed cell death such as detachment, shrinkage and blebbing are observed. **(b)** For a better appreciation of nuclear events such as nuclear condensation and fragmentation, cells exposed to particles as in **(a)** imaged in all channels (merge) are also imaged only in the Hoechst channel.

References

1. DeLoid, G., Cohen, J.M., Darrah, T., Derk, R., Rojanasakul, L., Pyrgiotakis, G., Wohlleben, W., Demokritou, P., 2014. Estimating the effective density of engineered nanomaterials for in vitro dosimetry. Nat. Commun. 5, 3514.
2. DeLoid, G.M., Cohen, J.M., Pyrgiotakis, G., Pirela, S.V., Pal, A., Liu, J., Srebric, J., Demokritou, P., 2015. Advanced computational modeling for in vitro nanomaterial dosimetry. Part Fibre Toxicol. 12, 32.

3. Hsiao, I.L., Fritsch-Decker, S., Leidner, A., Al-Rawi, M., Hug, V., Diabaté, S., Grage, S.L., Meffert, M., Stoeger, T., Gerthsen, D., Ulrich, A.S., Niemeyer, C.M., Weiss, C., 2019. Biocompatibility of Amine-Functionalized Silica Nanoparticles: The Role of Surface Coverage. Small e1805400.
4. Leibe, R., Hsiao, I.L., Fritsch-Decker, S., Kielmeier, U., Wagbo, A.M., Voss, B., Schmidt, A., Hessman, S.D., Duschl, A., Oostingh, G.J., Diabaté, S., Weiss, C., 2019. The protein corona suppresses the cytotoxic and pro-inflammatory response in lung epithelial cells and macrophages upon exposure to nanosilica. Arch. Toxicol. 93, 871-885.
