# Supplementary material for: Silica Nanoparticles Provoke Cell Death Independent of p53 and BAX in Human Colon Cancer Cells
Source: Nanomaterials (Basel). 2019 Aug 16;9(8):1172. doi: 10.3390/nano9081172 (PMC6724124; doi:10.3390/nano9081172)
Supplement: Supplementary file 1 [file nanomaterials-09-01172-s001.zip › Video-S1_Nanomaterials.pptx]

## Slide 1
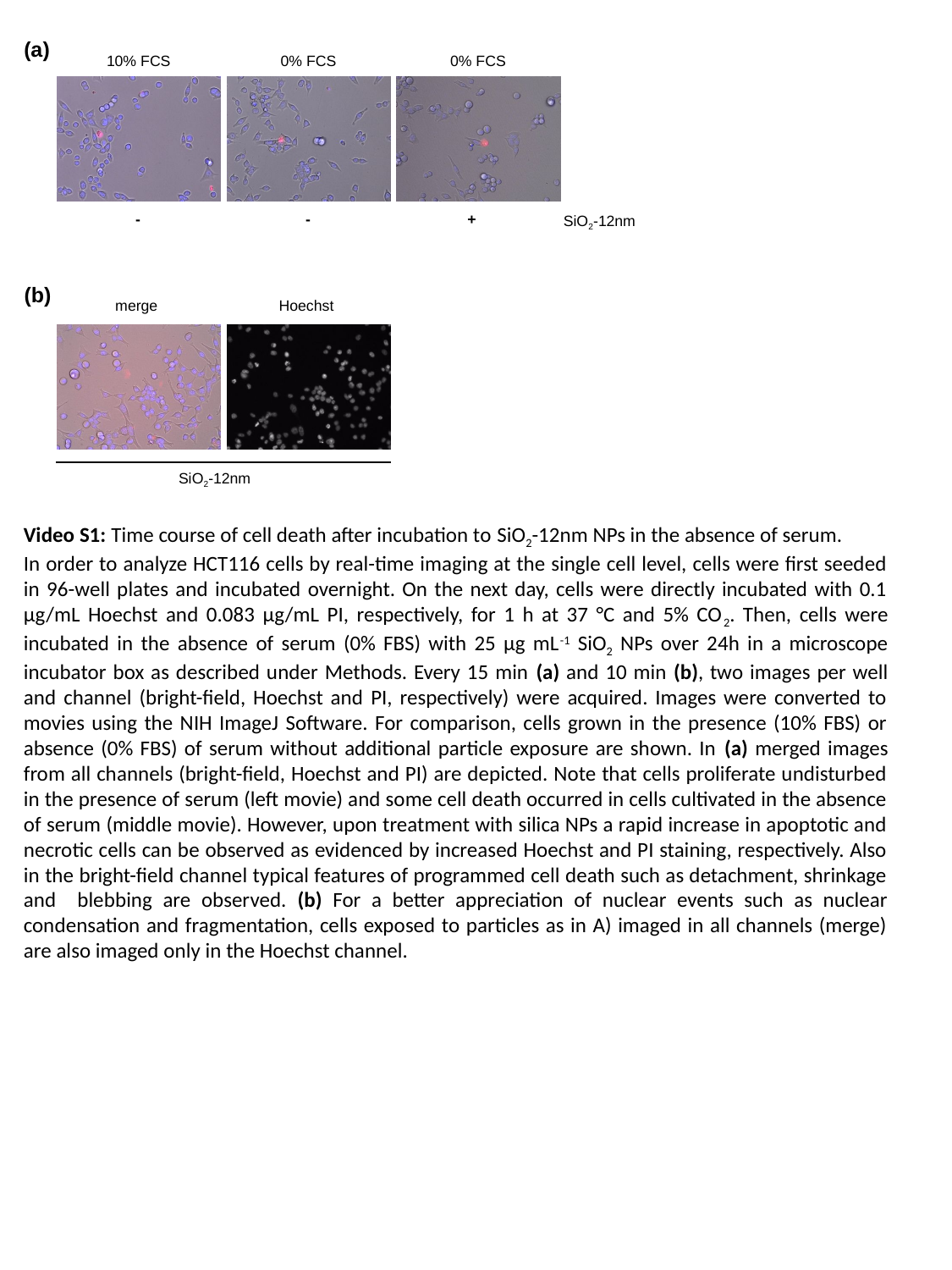

(a)
10% FCS
0% FCS
0% FCS
-
-
+
SiO2-12nm
(b)
merge
Hoechst
SiO2-12nm
Video S1: Time course of cell death after incubation to SiO2-12nm NPs in the absence of serum.
In order to analyze HCT116 cells by real-time imaging at the single cell level, cells were first seeded in 96-well plates and incubated overnight. On the next day, cells were directly incubated with 0.1 μg/mL Hoechst and 0.083 μg/mL PI, respectively, for 1 h at 37 °C and 5% CO2. Then, cells were incubated in the absence of serum (0% FBS) with 25 µg mL-1 SiO2 NPs over 24h in a microscope incubator box as described under Methods. Every 15 min (a) and 10 min (b), two images per well and channel (bright-field, Hoechst and PI, respectively) were acquired. Images were converted to movies using the NIH ImageJ Software. For comparison, cells grown in the presence (10% FBS) or absence (0% FBS) of serum without additional particle exposure are shown. In (a) merged images from all channels (bright-field, Hoechst and PI) are depicted. Note that cells proliferate undisturbed in the presence of serum (left movie) and some cell death occurred in cells cultivated in the absence of serum (middle movie). However, upon treatment with silica NPs a rapid increase in apoptotic and necrotic cells can be observed as evidenced by increased Hoechst and PI staining, respectively. Also in the bright-field channel typical features of programmed cell death such as detachment, shrinkage and blebbing are observed. (b) For a better appreciation of nuclear events such as nuclear condensation and fragmentation, cells exposed to particles as in A) imaged in all channels (merge) are also imaged only in the Hoechst channel.
